# Sub-minimum inhibitory concentration of rifampin: a potential risk factor for resuscitation of *Mycobacterium tuberculosis*

**DOI:** 10.1186/s13756-017-0273-1

**Published:** 2017-11-14

**Authors:** Shahin Pourazar Dizaji, Alireza Taala, Morteza Masoumi, Nayereh Ebrahimzadeh, Abolfazl Fateh, Seyed Davar Siadat, Farzam Vaziri

**Affiliations:** 1 Department of Mycobacteriology and Pulmonary Research, Pasteur Institute of Iran, Tehran, Iran; 2 Microbiology Research Center (MRC), Pasteur Institute of Iran, Tehran, Iran

**Keywords:** *Mycobacterium tuberculosis*, Resuscitation promoting factors, Rifampin, Sub MIC, Transcriptional profile

## Abstract

**Background:**

*Mycobacterium tuberculosis* possesses five resuscitation-promoting factors, Rpf A to E, which are required for the resuscitation of dormancy in mycobacteria. This study explores the transcriptional profile of all five *rpfs* of *M. tuberculosis,* in response to sub-MIC concentration of rifampin, in multidrug and mono-rifampin resistant clinical isolates.

**Methods:**

Thirteen multidrug and two rifampin mono resistant clinical isolates were analyzed. Drug susceptibility testing and determination of MIC were performed. The relative expression of *rpfs* was measured, by real-time quantitative PCR.

**Results:**

A significant upregulation of relative expression (*p* < 0.05) was observed, as follows: 7/15(46.66%); 5/15(33.33%); 9/15(60%); 10/15(66.66%) and 9/15(60%) in *rpfA*, *rpfB*, *rpfC*, *rpfD* and *rpfE*, respectively.

**Conclusion:**

Our results showed that the *rpfs* could be overexpressed in some extent in the presence of sub-MIC concentration of rifampin in multidrug and mono drug resistant *M. tuberculosis*. These results highlight the potential risk of sub-MIC rifampin concentrations, as a risk factor for tuberculosis reactivation.

## Background

Tuberculosis (TB) is a life-threatening bacterial disease, caused by *Mycobacterium tuberculosis (M. tuberculosis)*. This bacterium is a very successful pathogen that can persist in its host, regardless of a strong immune response; and causes latent TB infection (LTBI) [1]. The situation will become more complicated, by the emergence and spread of multidrug-resistant (MDR-TB) and Rifampin mono resistant (RMR-TB) strains of *M. tuberculosis*, which represents a major challenge to the global control of the TB [2–4]. Although the host-pathogen interactions resulting in LTBI remain elusive, the exact mechanism through which *M. tuberculosis* switches from its dormant state to active form, is currently attracting much of the research activity in this field [5].

The factors found to be responsible for awakening the dormant bacteria, belong to the so-called Rpf (Resuscitation promoting factors) proteins. This family of proteins in *M. tuberculosis* is composed of Rpf A to E [6]. Rpfs have been proposed to act as peptidoglycan hydrolases, implicated in remodeling of the bacterial cell wall, through cleavage of the beta-1,4-glycosidic bond in peptidoglycan, which help in cell division and/or release of anti-dormancy factors [7]. Experiments with *M. tuberculosis rpf* mutant strains have shown that Rpf proteins determine the ability of such strains to establish the LTBI in animal models and to be resuscitated with the development of an active TB [8].

Expression of different factors in bacteria is regulated by a network of regulatory and signaling pathways that respond to specific environmental cues, such as pH, nutrient status, ions, temperature and most importantly, antibiotics at sub-Minimum Inhibitory Concentration (MIC), which can alter the expression pattern of the genes [9].

In the current study, an attempt has been made to prove this hypothesis that sub-MIC concentration of rifampin might be a potential risk factor in the resuscitation of MDR and RMR in *M. tuberculosis* clinical isolates.

## Methods

### Bacterial strains and drug susceptibility testing

Thirteen MDR and two RMR clinical isolates were selected, from *M. tuberculosis* strains collection of the Department of Mycobacteriology and Pulmonary Research, Pasteur Institute of Iran. *M. tuberculosis* strains were isolated from patients with pulmonary TB, from January 2010 to March 2015. Ethical reviews and informed written consent approval were granted by the Ethical Committee of the Pasteur Institute of Iran (Tehran, Iran). Informed consent was obtained from all of the patients, enrolled in the study. The isolate profiles of drug susceptibility were re-confirmed by the proportional method, using Lowenstein-Jensen medium, supplemented with Isoniazid (INH), 0.2 mg/L; Rifampin (RIF), 40 mg/L; Streptomycin (STR), 4 mg/L; Ethambutol (EMB), 2 mg/L; Kanamycin (KAN), 30 mg/L and Ofloxacin (OFX), 2 mg/L [10]. Bacterial growth on antimicrobial agents-containing media, exceeding 1 % of the number of colonies on antimicrobial agents-free media (control), were considered to be resistant to the antimicrobial agents. All experiments were performed, in accordance with the guidelines, approved by Pasteur Institute International networks.

### Determination of MIC

An Alamar blue assay (Thermoscientific, USA) was carried out, as previously described, to determine the MICs of RIF of the 15 clinical isolates [11]. RIF concentrations were 0.001–256 mg/L. All tests were conducted in duplicate. The MIC was defined as the lowest antibiotic concentration that inhibits any color change. Isolates with MICs of RIF <1 mg/L were defined, as being susceptible. For next-step analysis, the bacteria, growing in sub MIC concentration were selected.

### RNA extraction and reverse transcription

All *M. tuberculosis* strains were sub-cultured in 7H9 medium, supplemented with Albumin Dextrose Catalase (ADC), in the absence of rifampin (control strains) and presence of sub-MIC of rifampin (MIC = 128 mg/L), and collected after four weeks for RNA extraction. Total bacterial RNA was isolated, using PREP-NA DNA/RNA extraction kit (DNA technology, Russia). The quality and integrity of the total RNA was assessed, using a nanophotometer (Thermoscientific, USA) and agarose gel electrophoresis. After DNase I treatment, RNA (1.5 μg) was reverse transcribed, according to the manufacturer’s recommendations (PrimeScript First Strand cDNA Synthesis Kit, Takara, Japan), and the thermal cycling conditions were as follows: 25 °C for 10 min, 42 °C for 60 min, and 85 °C for 5 min. The cDNAs were maintained at −20 °C, until further use.

### Quantification of gene expression using real-time quantitative PCR

The primers for *rpf (A-E)* and *16S rRNA* were described, previously [12]. The assay was performed, using a SYBR Premix Ex Taq™ II kit (Takara, Japan) in a LightCyler 96 thermocycler (Roche, Germany). Briefly, 10 μL qPCR mix, 0.8 pmol each primer, 10 ng cDNA, and RNase-free water were mixed, with a final volume of 20 μ L. The thermal cycling conditions were as follows: 95 °C for 1 min, then 40 cycles of denaturation at 95 °C for 10 s, annealing at 55 °C for 30 s, and extension at 72 °C for 30 s, and the last step consisted of a melting curve analysis (65–97 °C).

The fold change in the gene expression under rifampin stress in all isolates was calculated by 2^-ΔΔCT^ method [13]. *16S rRNA* is a housekeeping gene that is expressed at a stable level in the isolates and can be used, as an internal invariant control. For each assay, the results were compared with corresponding *M. tuberculosis* control growth, in the absence of the rifampin, as the calibrator. Each assay was performed in triplicate, for each gene.

### Statistical analysis

Two-tailed t-test was used for data analysis. All calculations were performed, using SPSS version twenty-two (SPSS Inc., Chicago, IL, USA). Lightcycler 96 software was used for expression data analysis. A *p* value of <0.05 was considered statistically significant.

## Results

Table 1 shows the antimicrobial resistance pattern for all antimicrobial agents used, and the MIC for RIF. Table 2 shows the relative quantification of the transcript levels for the five *rpfs* in *M. tuberculosis* isolates, exposed to sub-MIC (MIC = 128 mg/L) concentration of RIF.

**Table 1:** Antimicrobial resistance pattern of six antimicrobials and MIC of RIF

*M. tuberculosis* strains	INH	RIF	STR	EMB	KAN	OFX	MIC of RIF mg/L
1	S	R	S	S	S	S	256
2	S	R	S	S	S	S	256
3	R	R	R	R	S	S	256
4	R	R	R	S	S	S	256
5	R	R	R	S	S	S	256
6	R	R	S	S	S	R	256
7	R	R	R	R	S	R	256
8	R	R	S	R	S	S	256
9	R	R	R	R	R	S	256
10	R	R	R	R	R	S	256
11	R	R	R	R	S	R	256
12	R	R	R	R	R	R	256
13	R	R	R	R	R	S	256
14	R	R	R	R	R	S	256
15	R	R	R	R	S	R	256

*R* Resistant, *S* Susceptible, *INH* Isoniazid, *RIF* Rifampin, *STR* Streptomycin, *EMB* Ethambutol, *KAN* Kanamycin, *OFX* Ofloxacin

**Table 2:** Relative expression results of *rpf* genes under sub MIC of rifampin

*M. tuberculosis* strains	*rpfA*	*rpfB*	*rpfC*	*rpfD*	*rpfE*
1	**1.073** ^*^	0.765	**1.191** ^*^	**20.580** ^**^	0.973
2	0.451	0.746	**3.976** ^**^	**1.600** ^*^	0.838
3	**1.249** ^*^	0.650	0.637	**1.211** ^*^	**1.009** ^*^
4	**1.165** ^*^	0.633	**1.174** ^*^	**1.724** ^*^	**2.575** ^**^
5	0.493	**2.644** ^**^	**5.275** ^**^	**2.854** ^**^	**1.356** ^*^
6	0.767	0.760	0.901	**1.642** ^*^	**1.022** ^*^
7	0.567	0.956	**1.361** ^*^	0.790	0.970
8	0.781	**1.298** ^*^	0.498	**26.750** ^*^	**2.017** ^**^
9	**0.410** ^*^	**13.390** ^**^	**6.726** ^**^	**1.399** ^*^	**1.904** ^*^
10	**1.309** ^*^	**1.170** ^*^	**1.324** ^*^	0.641	0.771
11	–	**0.398** ^*^	**1.168** ^*^	0.752	0.932
12	0.727	**1.087** ^*^	0.671	**1.206** ^*^	**1.021** ^*^
13	**1.458** ^*^	0.986	0.655	0.978	**1.250** ^*^
14	**1.432** ^*^	**0.385** ^*^	**1.542** ^*^	**1.344** ^*^	**1.198** ^*^
15	**1.738** ^*^	0.789	0.475	0.834	0.665

Bold ratio: *Statistically significant; *P* < 0.05, Relative expression compared with corresponding *M. tuberculosis* control growth in the absence of the rifampin

Bold ratio: **Statistically highly significant; *P* < 0.001, Relative expression compared with corresponding *M. tuberculosis* control growth in the absence of the rifampin

-: qPCR failure

Significant upregulation of relative expression (*p* < 0.05) was observed as follows: 7/15(46.66%), 5/15(33.33%), 9/15(60%), 10/15(66.66%) and 9/15(60%) in *rpfA*, *rpfB, rpfC, rpfD* and *rpfE,* respectively. Additionally, in three isolates (3/15; 20%) one *rpf*; four isolates (4/15; 26.66%) two *rpf*; five isolates (5/15; 33.33%) three *rpf* and three isolates (3/15; 20%) four *rpf* were upregulated, simultaneously. None of the isolates had all five-rpf upregulated, simultaneously (Table 2).

## Discussion

TB remains a significant public health problem, globally. The Rpf protein, if appropriately folded, is necessary for both cell wall hydrolysis and reactivation of *M. tuberculosis* [14]. The Rpfs are the secretory proteins, responsible for awaking bacilli from the persistent state*,* but the exact mechanism of regulation is yet to be determined [15]. Currently, the state of balance between replicating and dormant *M. tuberculosis,* during the course of infection from LTBI to active TB, remains theoretical. In one theory, a few dormant strains (have been termed ‘scouts’) resuscitate, through signals such as Rpfs. In the not well-defined situations, scouts send activation signals (most probably Rpfs) to the majority of the dormant bacteria, causing resuscitation [16, 17]. Understanding the physiology of *M. tuberculosis* during metabolic switching, among active-dormant phases is an interesting area of research.

Reactivation of LTBI is a major problem, in regards to the prevention of the spread of TB. Despite the importance of studying reactivation, limited progress has been made in our understanding of the events, during the transition. One scenario can be the effects of sub-MIC drug exposure on bacterial physiology. Depending on the bacterial species and the classes of antibiotic, these changes lead in either overexpression or downregulation of specific virulence gene, and it is often difficult to identify the exact underlying physiological mechanism, for such alterations [9]. Therefore, in the current study, we investigated the effect of sub-MIC concentration of rifampin on Rpfs expression, in resistant *M. tuberculosis* clinical isolates. It is noteworthy to mention that based on our analysis on H37Rv (a susceptible reference strain), no changes in the expression level of *rpfs* were detected (data not shown). This might indicate that very low MIC of RIF for this strain (and of course for other susceptible isolates), cannot affect the *rfp* expression. Therefore, we have decided to work on MDR and RMR isolates with high MIC. Our results showed that the *rpfs* could be overexpressed in some extent in the presence of sub-MIC concentration of rifampin in MDR and RMR *M. tuberculosis,* regardless of their antibiotic resistance pattern.

Some factors increase the risk of TB reactivation, and require screening and treatment for LTBI [18]. Our result highlights the potential risk of sub-MIC rifampin concentrations, as another risk factor in this regard. This issue may be more important in the treatment of LTBI by rifampin monotherapy [19].

Rpf-dependent mycobacteria appear to underlie the difficulty we have, in improving TB treatment, yet their presence appears to be driven, by both treatment and host factors [20]. It is noteworthy to mention that Rpf-dependent *M. tuberculosis* bacilli from sputum have been proven to be more tolerant to rifampin [21, 22]. Moreover, Hu et al. have recently demonstrated that the abundance of these Rpf- dependent *M. tuberculosis* bacilli, correlated with TB reactivation [23]. The correlation between these studies and our results needs further investigation.

Loraine et al. recently showed that Rpf overexpression rescues non-culturable mycobacteria, generated by treatment with first-line antimicrobial agents in vitro*.* In addition, Loraine et al. proved that the RpfD-overexpressing *M. smegmatis* strains showed consistently higher resuscitation, compared to the other Rpfs [24]. Interestingly, *rpfD* was the most prevalent (66.66%) and overexpressed gene in our study (Table 2). Collectively, Rpf overexpression can be a potential risk factor for unsuccessful TB treatment. On the other hand, we also observed downregulation (mostly not statistically significant) in the *rfps*, in response to sub MIC rifampin concentrations. Tufariello et al. showed that the *rpfs* had decreased expression during reactivation, compared to the chronic phase of TB [25]. However, most of the studies proved that the Rpfs are overexpressed, during resuscitation [26]. Most importantly, T cell responses may be a signal for bacteria to remain dormant, due to not only proper immune responses but also to the possible destruction of the Rpf proteins. In other words, a debilitated immune system, lead to increase in the Rpfs protein level, which may signal the bacteria to resuscitate [27]. So, overexpression of Rpfs can have similar effects.

Significant progress has been made to understand the biology of Rpfs, illuminating the pivotal role they play, in the TB pathogenesis [20]. According to our results, the sub-MIC rifampin concentrations may have important effects not only on the Rpf expression, but also in the pathogenesis of this ancient scourge (e.g.*,* by upregulation of *rpfs*). More investigation is inevitable to prove this hypothesis.

## Conclusions

In conclusion, while the clinical significance of Rpf overexpression awaits further evaluation, our findings indicate that the sub-MIC rifampin concentrations may have important effects on Rpf expression (at least at the transcriptional level), which may result in clinically relevant changes in bacterial behavior. This phenomenon must be investigated in a larger number of strains, using in vivo models in order to elucidate more details about its clinical importance in treatment of TB.
